# An Application of an Embedded Model Estimator to a Synthetic Nonstationary Reservoir Model With Multiple Secondary Variables

**DOI:** 10.3389/frai.2021.624697

**Published:** 2021-04-15

**Authors:** Colin Daly

**Affiliations:** Schlumberger Ltd., London, United Kingdom

**Keywords:** geostatistics, machine learning, random forest, Ember, spatial statistics, reservoir modeling

## Abstract

A method (Ember) for nonstationary spatial modeling with multiple secondary variables by combining Geostatistics with Random Forests is applied to a three-dimensional Reservoir Model. It extends the Random Forest method to an interpolation algorithm retaining similar consistency properties to both Geostatistical algorithms and Random Forests. It allows embedding of simpler interpolation algorithms into the process, combining them through the Random Forest training process. The algorithm estimates a conditional distribution at each target location. The family of such distributions is called the model envelope. An algorithm to produce stochastic simulations from the envelope is demonstrated. This algorithm allows the influence of the secondary variables, as well as the variability of the result to vary by location in the simulation.

## Introduction

An issue that practitioners of spatial modeling confront is that the ensemble behavior of the modeled random function might not match observable properties of the dataset in some types of application. For example, some auxiliary hypotheses of a generative model such as stationarity, needed to allow inference, may leave an unwelcome trace in the predicted results. The subsurface reality itself is rarely stationary, and the usual remedy for scientists is to subdivide the domain of interest into regions which they believe to be approximately stationary (or reducible to stationary by removing a trend) and to create separate models within each region. While this generally works well in practice, it does suffer from a couple of potential downsides. Sometimes the number of regions required can be quite large, necessitating a labor-intensive and quite error-prone procedure. Secondly, a region which is modeled as stationary may, in fact, have some subtle but distinct sub-regions which, if identified, would affect some desired applications of the model (for example, fluid flow strongly depends on vertical spatial divisions).

A compounding factor is that when predicting a target variable at a location, there often are a number of secondary variables known at that location which are covariate with the target variable of interest, e.g., Wackernagel (2003). An example from the oil industry is the prediction of porosity away from well locations, where the porosity value is known to a reasonably high degree. At target locations, we may have observed one or more seismic and/or geological attributes, which are covariate with porosity. These are known as secondary variables in Geostatistics. An observable spatial trend when seen in the data is often modeled as a polynomial. In many generative models, such as Gaussian cosimulation, a simplified relationship between the covariates and the target variable is assumed. For example, stationarity of the correlation between secondary variables and the target variable is an auxiliary hypothesis that is unlikely to be fully satisfied in practice.

In this article, a simple alternative procedure is proposed which is aimed at reducing these effects. Influenced by the idea of Conditional Random Fields (CRFs) and the classic Geostatistical wariness of very highly parametric models, the idea is to directly estimate an envelope of conditional distributions based on the secondary data and on prior speculative spatial models. The envelope of distributions can be thought of as a generalization of a trend model. They can be quite nonstationary, reflecting local heterogeneity patterns. They provide estimates of the target variable, as well as a locally adapted estimate of uncertainty. This can be carried out without invoking a full multivariate distribution. Unfortunately, it is not possible to extend the workflow to produce realizations of a random function without such an assumption. In most traditional formulations, this is made up front. Examples are Gaussian Random Fields (GFs), Markov Random Fields (MRFs), Hidden Markov Models (HMMs), and many variants. Since the prior model is generally made with some hypothesis of stationarity, the risk of this hypothesis persisting into the results should be considered.

For the approach considered here, the realizations are made by a conditional sampling from the distributions that we have estimated. However, it is only the sampling that needs to be assumed as stationary. Hence, the fully spatial model is only for the sampling function, and there is no explicit random function model of the target variable itself, only an envelope of distributions which depend on the state of knowledge about the target. To summarize,

(a) A machine learning/conditional distribution estimation algorithm is used to get a distribution at each spatial location. This family of distributions is called the envelope in this article.
(b) A stationary ergodic random function is used to sample from the envelope at each location conditionally on the observed value of the target variable at that location.

As well as being the basis for simulating realizations, the envelope of conditional distributions can be used to obtain estimates of the mean, quantiles, robust estimates of uncertainty, and unbiased estimates of “sweet spots” such as locations where porosity is expected to be higher than a user set threshold. The idea of “embedding” prior spatial models in the estimation of the envelope is what gives rise to the name Ember, which stands for embedded model estimator. This allows use of an essentially unlimited number of additional variables to enhance conditioning of the final model. It turns out to still be possible to make inference about the type of variogram to use for the stationary sampling RF depending on the assumptions about the type of sampling that we wish to use. A major advantage of this method is that the final estimates are now allowed to be nonstationary. In other words, the predictor may “reweight” the importance of the variables in different parts of the field to adapt to local heterogeneity patterns.

After a more technical introduction to the method, an application to a synthetic subsurface reservoir model is shown. Additional technical discussion of the algorithm is held back to the appendix. Some alternative examples are in Daly, 2020, a more technically focused paper.

## Embedded Model Decision Forests

In the classical universal kriging model, the trend is a point property (i.e., there is one value at each target location) and is often considered to be an estimate of the mean or of the low-frequency component of the model. It is not exact, in the sense that it does not honor the observed values of the target variable. Typically, it is constructed either as a local polynomial of the spatial co-ordinates (universal kriging) or using some additional variable(s) (e.g., external drift). In the Ember model, a conditional distribution is estimated at each location. In an analogy with the trend, the conditional distribution is built using the spatial co-ordinates and additional variables. In addition, the envelope estimation step will often use the predictions of simpler models to aid calculation of the conditional distributions at each location by embedding. In this work, the embedded model is kriging. The rationale is that the secondary variables, which might include seismic attributes, stress estimates, distance to the nearest fault, and height above contact, as well as true vertical depth, stratigraphic depth, and x,y spatial locations, do not explicitly contain information about the spatial correlation which is available in kriging through the variogram. In the example, we will see that including the additional information which kriging brings can help constrain the envelope. Depending on the case, it may be a weak variable, contributing little, or a very strong variable which comes close to determining the solution. We note that embedding models will take a little extra work as models do not behave exactly the same as data in training.

Conditional Random Fields (CRFs) avoid construction of the multivariate law (Lafferty et al., 2001). The advantage in direct estimation of each conditional distribution in the envelope compared to a generative Bayesian model is that no effort is expended on establishing relations between the numerous predictor variables. In a full spatial model, these involve stringent hypothesis such as the stationarity of the property of interest (perhaps coupled with some simple model of trend) and the stationarity of the relationship between the target variables and the explanatory variables (e.g., the hypothesis that the relationship between porosity and seismic attributes does not change spatially). The principle impact of stationarity in the classic model is seen in stochastic realizations which need to invoke the full multivariate distribution and, therefore, lean heavily on the hypotheses. This can be greatly reduced in the current proposal.

The form of CRF that we use here to calculate the envelope accommodates and embeds existing spatial models using a Markovian hypothesis. Let Z(x) be a target variable of interest at the location x, and let Y(x) be a vector of secondary or auxiliary variables observed at x. Let $\left\{Z_{i},\;Y_{i}\right\}$ be observations of the target and secondary variables observed in the field, i.e., $Z_{i}$ denotes the value of the target variable $Z\left(x_{i}\right)$ at training location $x_{i}$. Finally, let $M\left(x\right)\;=\;f\left(\left\{Z_{i},\;Y_{i}\right\}\right)$ be a vector of pre-existing estimators of Z(x). Then, the form of CRF that we require is that the conditional distribution of Z(x) given all available data $\hat{F}\left(z\text{|}Y\left(x\right),\;\left\{Z_{i},\;Y_{i}\right\}\right)$ satisfies

$$\hat{F}\left(z\text{|}Y\left(x\right),\;\left\{Z_{i},\;Y_{i}\right\}\right)=\;E\left[1_{Z\left(x\right)<z}|Y\left(x\right),\;\left\{Z_{i},\;Y_{i}\right\}\right]\approx E\left[1_{Z\left(x\right)<z}|Y\left(x\right),M\left(x\right)\right]\;.$$ (1)

This hypothesis states that the conditional distribution of Z(x) given all the secondary values observed at x and given all the remote observations of $\left\{Z_{i},\;Y_{i}\right\}$ can be reduced to the far simpler conditional distribution of Z(x) given all the secondary values observed at x and the vector of model predictions at x. The focus is now on trying to estimate the right-hand side of Eq. 1 at each location. Notice that Eq. 1 is not an exact Markov hypothesis. There is a loss of information. In particular, using M(x) merely as an “oracle” that makes predictions at x means that the estimated conditional distribution does not collapse to a singularity at the data locations. As already said, the envelope resembles a trend and conditional simulations will require a conditional sampling from it.

In this work, we choose to base the algorithm for calculation of the envelope on a highly successful nonparametric paradigm, the Decision Forest, e. g., Breiman (2001), Breiman (2004), Breiman (1996), Meinshausen (2006), Athey et al. (2019), Ho (1998), Lin and Jeon (2006), Mentch and Zhou (2019), and Scornet (2015). An introduction to the method can be found in the Supplementary Material. A decision forest is a set of decision trees, see Breiman, Friedman et al. (1984) and Györfi et al. (2002). For the training stage, each tree starts with a single node containing all the training data. For now, let us ignore the role of the embedded models. The training data are, therefore, vectors $\left(Z_{i},Y_{i}\right)$. Each node is split reclusively using a threshold on one of the variables $Y_{k}$, for some k, in a way that helps to improve the fit until the terminal nodes contain (typically) a single sample. To predict at a location, Z(x), with secondary vector Y(x)=y for the single tree, the value of y is “dropped” down the tree, and the prediction of Z(x) is the value of $Z_{i}$ found in the terminal node where y ends up. Each tree in the forest is generated with some random parameters meaning that the predicted result can change from one tree to the next. The final estimator of the conditional distribution is of the form

$$\hat{F}\left(z\text{|}Y\left(x\right),\;\left\{Z_{i},\;Y_{i}\right\}\right)=\;\overset{n}{\underset{i=1}{\sum }}\omega_{i}\left(y\right)1_{\left\{Z_{i}<z\right\}},$$ (2)

where the weights $\omega_{i}\left(y\right)$ count how frequently the value of $Z_{i}$ is used as a predictor. Under certain conditions, it can be proved that $\hat{F}\left(z\text{|}Y=y\right)\to F\left(z|Y=y\right)$ as n→∞ (Supplementary Material).

Embedded models are treated slightly differently to secondary data. They are embedded (Supplementary Material) by training our decision forest on cross-validated estimates. Thus, our training dataset for each tree is $\left\{Z_{i};\;Y_{i},M_{-i}\right\}$, where $M_{-i}\equiv M\left(x_{i}\right)$ are model estimates at training location $x_{i}$ which do not use the information available at $x_{i}$ (they are cross validated). With an estimate of the conditional distribution now available at every target location, it is a simple matter to read off estimates of the mean of this distribution, which we call the Ember estimate. When kriging is the strongest variable, the Ember estimate is usually close to the kriging estimate but will typically be better than kriging if the secondary variables are the strongest. It must be noted that the Ember estimate, unlike kriging, but in analogy with trend modeling, is not exact. It is also possible to quickly read off quantiles, measures of uncertainty such as spread, and P90-P10, as well as interval probabilities of the form P(a≤Z(x)≤b) .

A number of methods have been proposed to do this, for example using Projection Pursuit, Barnett et al. (2014). The idea here, influenced by the idea of the Cloud Transform, e.g., Kolbjørnsen and Abrahamsen (2004), is to model *Z* (*x*) by taking a conditional sample from the envelope of distributions F(Z(x)|Y(x)=y) using a uniform stationary, ergodic random field U(x), such that the result is conditioned at the hard data locations. If we use a transformed Gaussian random field for U(x)= G(X(x)), for a multi-Gaussian random field X(x), then this can be achieved by a Truncated Gaussian simulation Freulon (1992). Moreover, in this case, we can obtain a relationship between the experimental variogram for our target variable Z(x) and that of the sampling random function, allowing appropriate model fitting. An approximate version of this relationship when Z itself is close to Gaussian is

$$\rho \left(x_{1}-x_{2}\right)=E\left[\left(\frac{Z\left(x_{1}\right)-\hat{\mu }\left(x_{1}|y\right)}{\sigma_{x_{1}}}\right)\left(\frac{Z\left(x_{2}\right)-\hat{\mu }\left(x_{2}|y\right)}{\sigma_{x_{2}}}\right)\right],$$ (3)

where ρ(h) is the covariance function of the Gaussian RF, X(x), to be simulated, $\hat{\mu }\left(x|y\right)$ is the Ember estimate, and $\sigma_{x}$ is the standard deviation of the residual $Z\left(x\right)-\hat{\mu }\left(x|y\right)$ which can be read from the estimate of the conditional distribution at x. The proof is sketched in the supplementary material and in a bit more detail in Daly, 2020.

## Application

**FIGURE 1:**
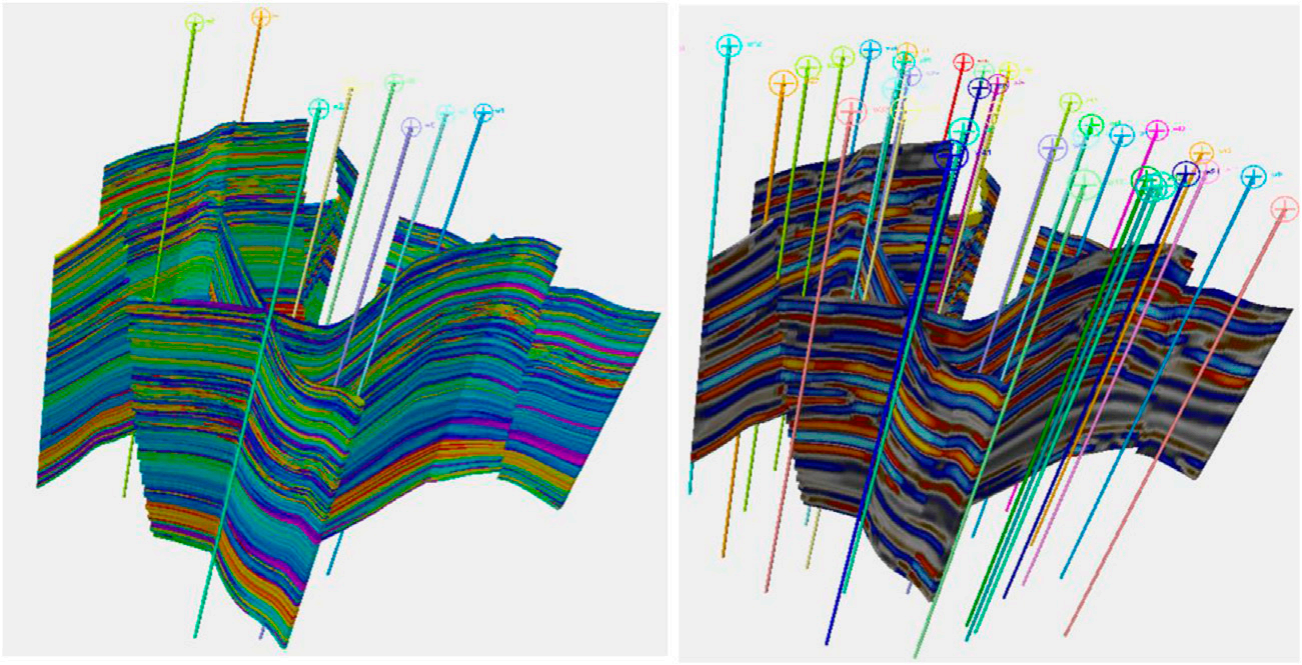
On the left, the true porosity of the reservoir to be modeled is shown. On the right, a synthetic RAI volume created is shown. The locations of the first eight wells are shown on the left, while the thirty-six-well case is on the right.

A synthetic reservoir was constructed to allow for exploration of some of the issues raised in realistic surroundings. For a real case study, see Daly et al., 2020. The model alternates between marine-dominated stacked shoreface sands in which the porosity varies quite smoothly, prograding in the distal direction which is to the southwest and fluvial systems which vary in terms of their net to gross. Figure 1 shows the “true” porosity in the reservoir and a Relative Acoustic Impedance (RAI) image. The true porosity is unknown and is the target variable to be estimated. We will consider two cases, one with few training data and the other with more data. These are 8 and 36 wells, respectively, and their positions are shown in Figure 1.

**FIGURE 2:**
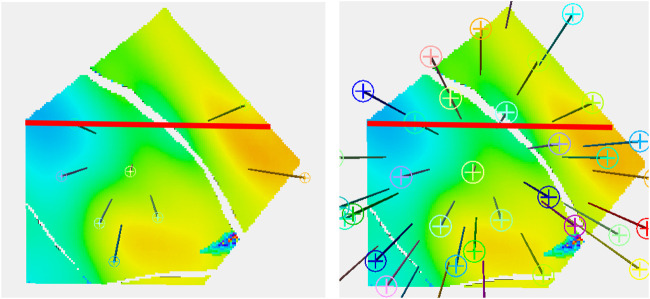
Plan view with the 36-well configuration on the right and the eight-well one on the left.

Figure 2 shows the wells on a plan view of the reservoir in one of the shoreface layers. In the eight-well case, six of the wells are in the central fault block, with two wells in the block to the north-east where the best porosity for shoreface sands is usually to be found. There are no wells in the smaller block to the south-west. The 36-well case has a better distribution of well location. The thick red line on the images represent the location of the cross section that is used in many of the subsequent figures. In the eight-well case, there are two wells in quite close proximity to the section, one at the either end, but the center is not particularly well controlled by well information.

**FIGURE 3:**
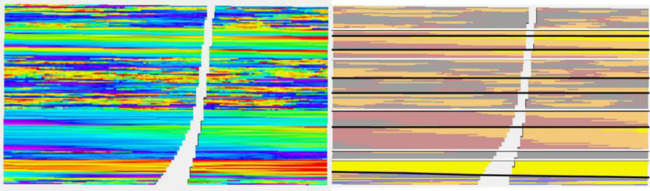
Cross section of the reservoir. The facies used for model construction are shown on the right, although these are not used in subsequent modeling effort.

**FIGURE 4:**
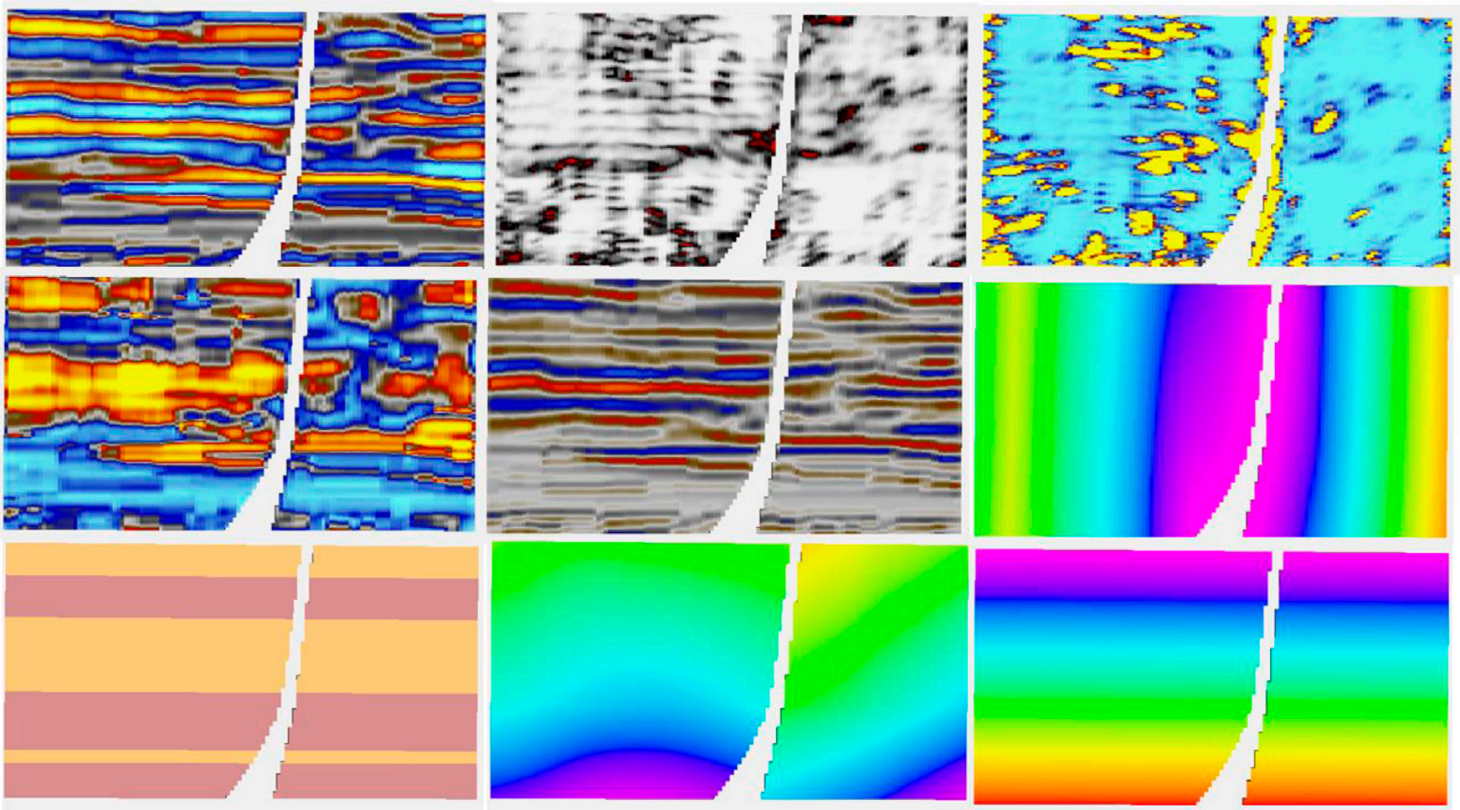
Cross section of nine of thirteen data variables used in modeling. From L to R per row: RAI, chaos, flatness: sweetness, amplitude, dist. to Fault: depositional zones, TVD, and strat. depth.

A synthetic seismic volume was created from a slightly modified version of the reservoir to ensure that it does not correspond too neatly to the “real” reservoir. Several attributes were derived from this volume. The modeling was performed using 13 data variables and two embedded kriging models giving a total of 15 secondary variables. A cross section of the reservoir together with the facies distribution, which is not known for the study, is shown in Figure 3. The black lines mark the reservoir zones. The 13 data variables consisted of five seismic variables and eight geometric variables. Nine of the data variables are shown in Figure 4.

For simulations, a variogram is required for the Sampling Random Function. In many real-world reservoir modeling cases, there is not enough information to calculate reliable variograms, especially in the horizontal direction. In such cases, the range is considered an uncertainty. The same is true using the Ember model, although as we shall see, the uncertainty is often somewhat mitigated by the estimate of the conditional distribution. In the case that a meaningful fit for the variogram of the target variable can be found for classical modeling, then using Eq. 3, it is likely that there is also a route to find the variogram for the Sampling RF (see Daly (2020) for an example). Indeed, for the 36-well case, it was possible to obtain a reasonable estimate for the sampling RF’s variogram. This was not the case for the eight-well situation, so, as usual, users will need to consider robustness and uncertainty of the estimation and simulation with regard to a poorly defined variogram.

**FIGURE 5:**
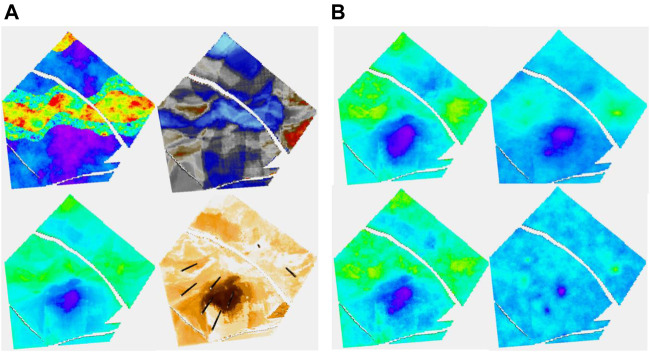
**(A, B)** Eight-well case. (A) Clockwise, truth, RAI, uncertainty spread (P90-P10), and prior mean; (B) clockwise, posterior Ember mean-good variogram (G.V), posterior Gaussian mean (G.V), posterior Gaussian mean-poor variogram (P.V), and posterior Ember mean (P.V).

Figure 5, which deals with the model of layer 91 in the case of having eight wells, has two parts, A and B. The four figures on the left are part A. It is a layer with quite a low net to gross. The channels are partly identified by the seismic. In 5A, the truth (i.e., the target porosity variable we are trying to estimate) and RAI, as well as estimates of the spread (P90–P10) and the mean of the estimated conditional distributions, are shown. With only eight wells, the variability of the mean estimate is low compared to the true distribution. Remember that the estimated mean does not exactly honor wells, though is often fairly close. We call this the prior mean.

Since Ember simulations condition to wells, we calculate a posterior mean by averaging many realizations. While it does not seem to bring much in terms of new information compared to the prior mean, and so probably does not need to be routinely calculated, the results of the posterior mean for Ember, as well as for Gaussian simulation (sometimes called the E-type), are shown in Figure 5B. To show the effect of using an incorrect variogram, the calculation is carried out twice, once using the exhaustive variogram and again with a model fitted to the empirical variogram. The variogram in the second case empirically fits the data quite well but is not a good match for the (unknown) true variogram (having only about 15% of the true range). Of the four estimates in 5B, the one using the Gaussian model with the incorrect variogram is by far the worst performing due to the distribution of variability in the Gaussian case being governed directly by the choice of variogram.

**FIGURE 6:**
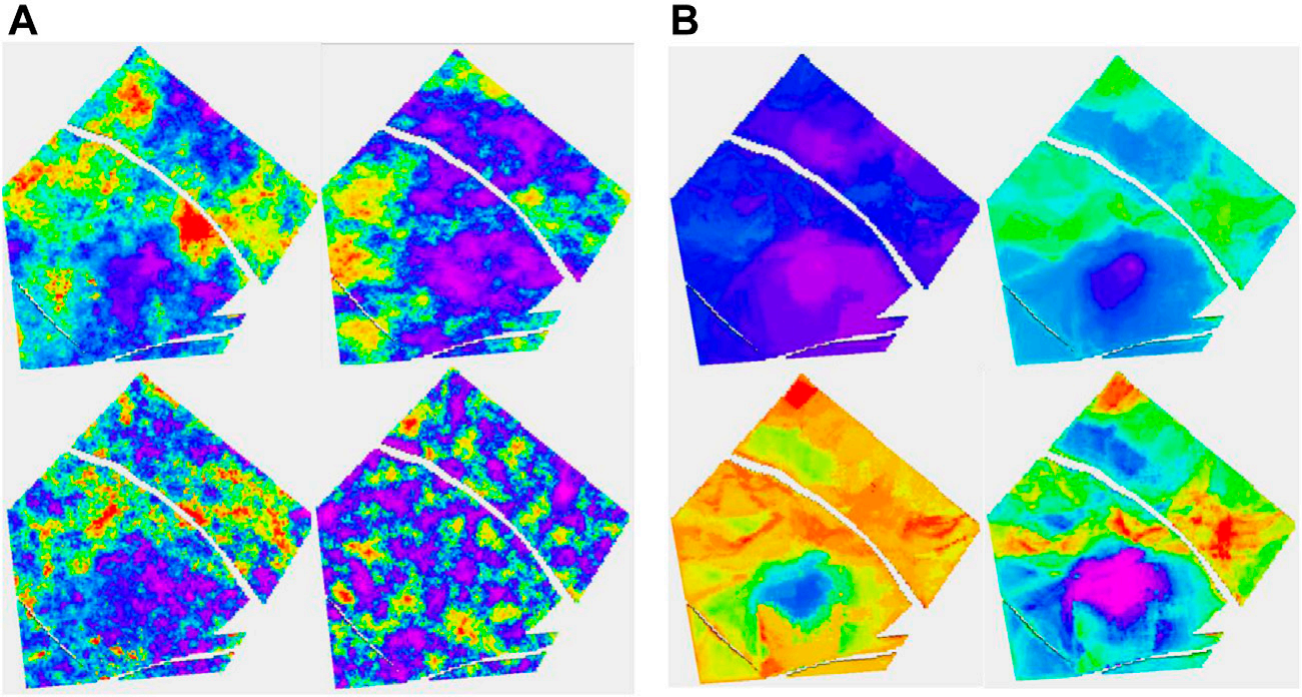
**(A, B)** Eight-well case. (A) Clockwise, Ember simulation-good variogram (G.V), Gaussian simulation (G.V), Gaussian simulation-poor variogram (P.V), and Ember simulation (P.V). (B) Clockwise, Ember quantiles: P10, P50, prob (porosity>20%), and P90.

**FIGURE 7:**
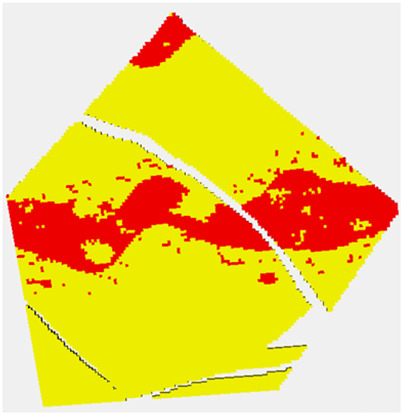
Red are locations when the true porosity is above 20%.

The results of stochastic simulation are shown in Figure 6A. The two figures at the top correspond to a simulation from Ember and from Gaussian simulation using the near optimal variogram, and the two below correspond to that using the short variogram model. Again, the relative homogeneity of the Gaussian short-range model is noticeable and is a result of the stationary hypothesis not being compatible with the true distribution, whereas the Ember result is far more robust. The figure on the right shows some by-products of the estimation phase of Ember modeling. These are the three quantiles, P10, P50, and P90, as well as the estimate of finding sand with high porosity of 20% or above. The true location of sand above 20% is shown in Figure 7 in red.

These high values lie within the channel belt. The channel belt is readily identified in the P50 and P90 cases, as well as the probability map. For context, the color red in the probability map corresponds to estimated probabilities of 0.5 or above of finding sand with porosity above 20%. The spread between P10 and P90 values show that while the channel is identifiable, there is still variability and so high porosity patches are still possible outside the belt.

**FIGURE 8:**
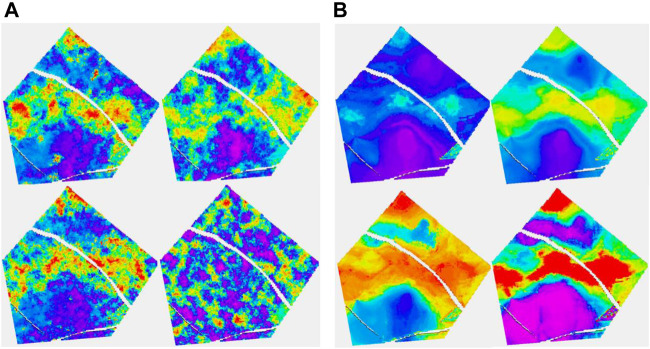
**(A, B)** Thirty-six well case. **(A)** The configuration follows that of Figure 6. **(B)** Top row is P10 and P50; bottom row is P90 and Prob (porosity>20%).

Turning attention to the 36-well case to get a feel for how additional information changes the Ember estimates, Figure 8 shows the same simulations and quantiles shown in Figure 6 with the training using the extra wells. The improvement is quite clear in the results. The additional information identifies and isolates the belts themselves quite well but is not quite enough to determine internal heterogeneity. This is simply a function of the geology as can be seen in Figure 13 which shows uncertainty of the Ember model in cross section. It is noticeable in Figure 8 that the distribution of heterogeneity still depends strongly on the variogram for the classical Gaussian models even with the increased well count and gives poor results for the short variogram.

**FIGURE 9:**
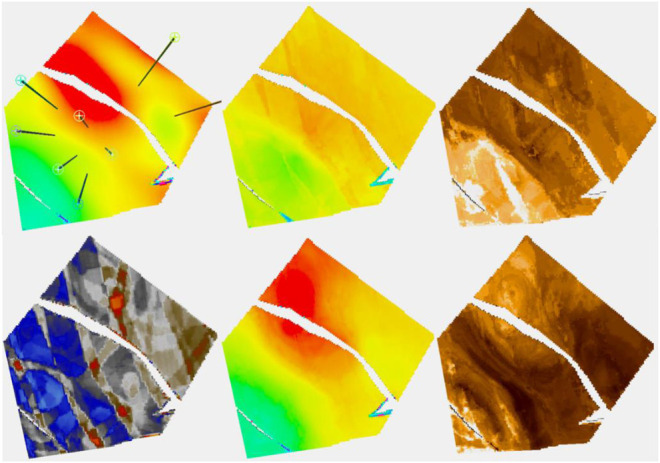
Upper images are the eight-well case, while the lower ones are the 36-well case. On the left is Ember simulation, while on the right is the associated uncertainty.

**FIGURE 10:**
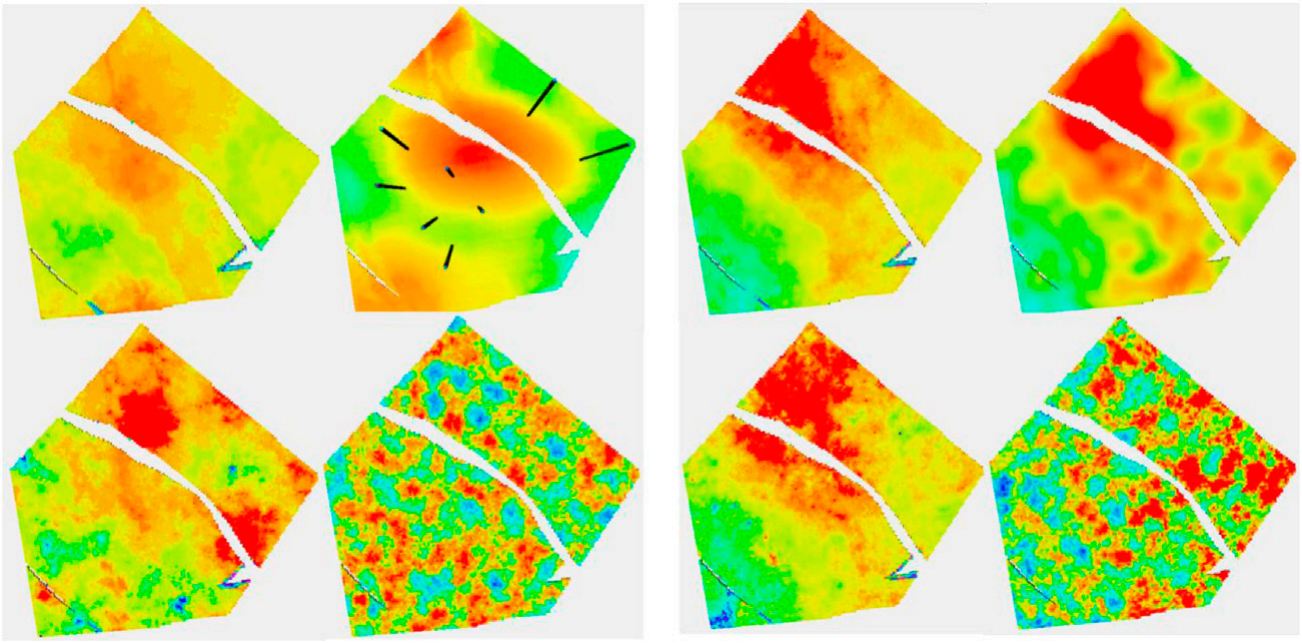
The classic Gaussian simulations referred to in Table 1. They differ by the trend management and the variogram used.

Next, a layer in the shoreface sands is considered. Uncertainty is lower in the shoreface, and moreover, it reduces more quickly with increasing data due to the greater simplicity. The porosity distribution is not well identified on seismic within the shoreface, so the Ember estimate is largely depending on geometric variables and the embedded kriging. Figure 10 focuses on the Ember estimation. As well at the truth and RAI, it shows the estimated prior mean for the 8- and 36-well cases, as well as their uncertainty estimates. Note that the patch of high porosity sand is not identified in the eight-well case but is in the 36-well case, as none of the initial eight wells sample it. The overall trend is acceptable even with eight wells.

**FIGURE 11:**
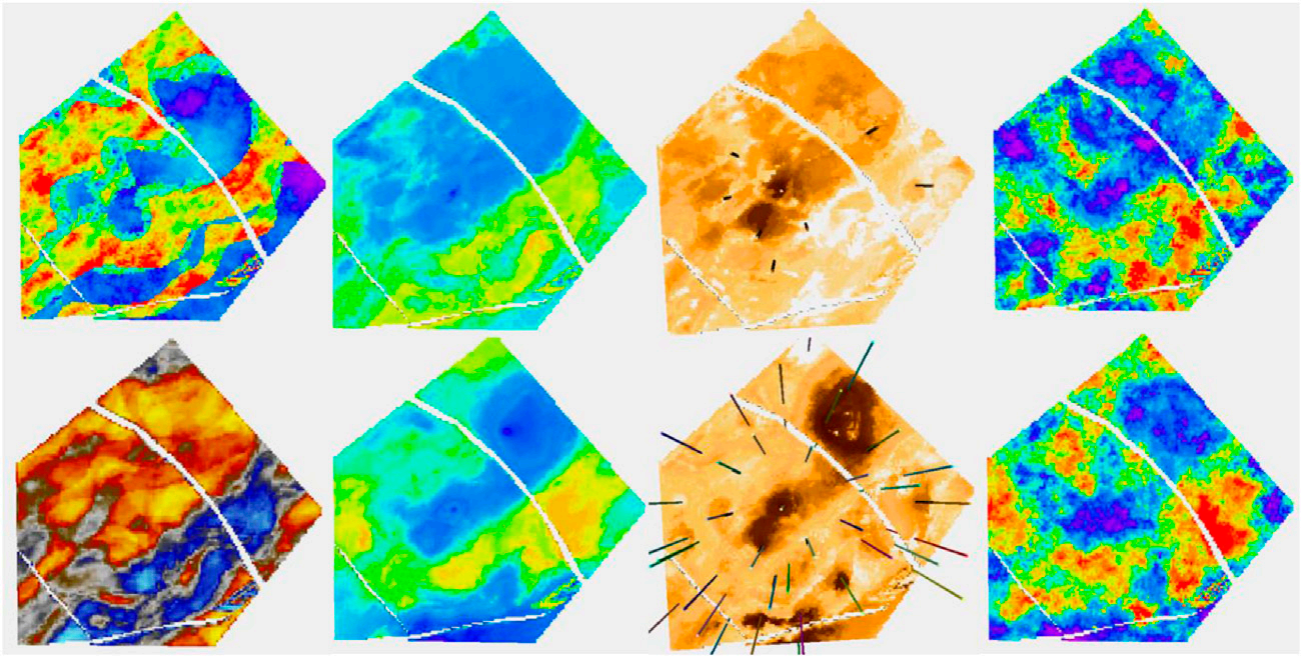
Ember results for a blind well, circled in red. Two layers are on the left, one in channel sand and the other in the shoreface. Their depth locations on the well track are shown with arrows. Tracks are, from left, true porosity of the blind well; prob (poro>15%); the next five tracks are simulations; true porosity (red) superimposed on envelope; and RAI.

Ember and Gaussian simulations are shown in Figure 11. As mentioned before, it is noticeable that the classic Gaussian model is less robust to an incorrect choice of variogram.

**FIGURE 12:**
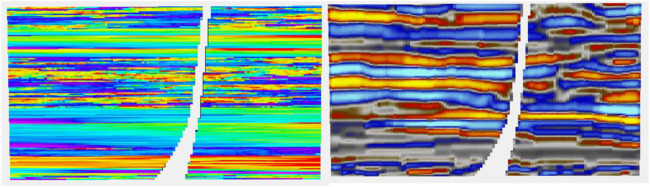
Same result as in Figure 15, with short-range variogram. Ember is shown on the left. Gaussian is shown on the right. Notice that the Gaussian envelope is almost uninformative compared to Ember.

In the two layers shown so far, the seismic was a large contributor for the channel case but played little role in the shoreface case. To show an intermediate situation, another layer from a channelized formation is shown in Figure 12. For this layer, we just look at the Ember solution as the Gaussian one performs similar to the previous case.

In the top left of Figure 12, two channel belts are visible, but only the easternmost one of them is readily discernible on the RAI seismic attribute. None of the eight wells traverse the thin western channel belt, although one well goes through the overbank. Only one well goes through the thicker eastern belt. The estimate of the mean for the eight-well case fails to identify the western belt, particularly to the north, but thanks to the seismic, it identifies the eastern one. The same is true for the simulation. The uncertainty is sufficient to allow some sand to be located in the northern part of the western channel belt in some realizations, but it is far from systematic and with the sampling random function (SRF) being Gaussian (and hence high entropy) is unlikely to form a connected structure in any case. This simple case shows that, in some cases, it may be of interest to look at the possibility of using lower entropy SRF to provide genuinely different Ember realizations or to use Ember to produce facies probabilities and continue with standard facies modeling methods.

In the 36-well case, both channels are well identified in the mean and the simulations tend to respect them. The eastern channel is still better identified due to the combination of seismic and embedded kriging, while the western one is less defined as it is less able to exploit the seismic attribute.

**FIGURE 13:**
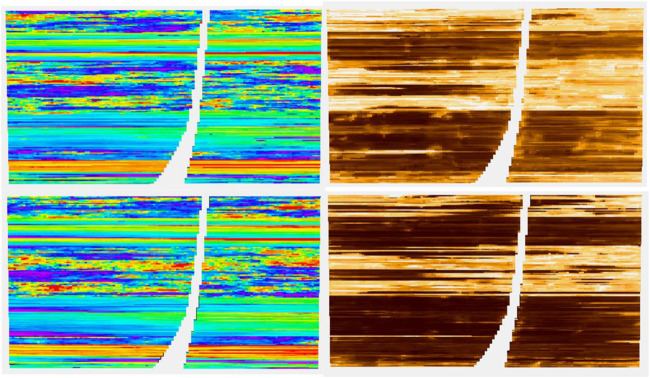
Shoreface layer. Left column, top truth and bottom RAI; middle column, Ember estimates and eight-well case on the top; right column, Ember uncertainty and eight-well case on the top.

**FIGURE 14:**
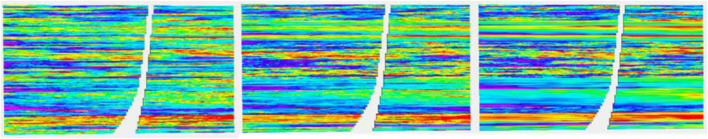
**(A)** For eight wells; **(B)** For 36 wells. Each group is organized as mentioned before, Ember on the left, the Gaussian model on the right, correct variogram on the top, and short variogram below.

So far, we have focused on plan views of several layers of the model. For completeness and to see the value of the additional well information, we return to the cross-sectional view in Figures 13, 14.

**FIGURE 15:**
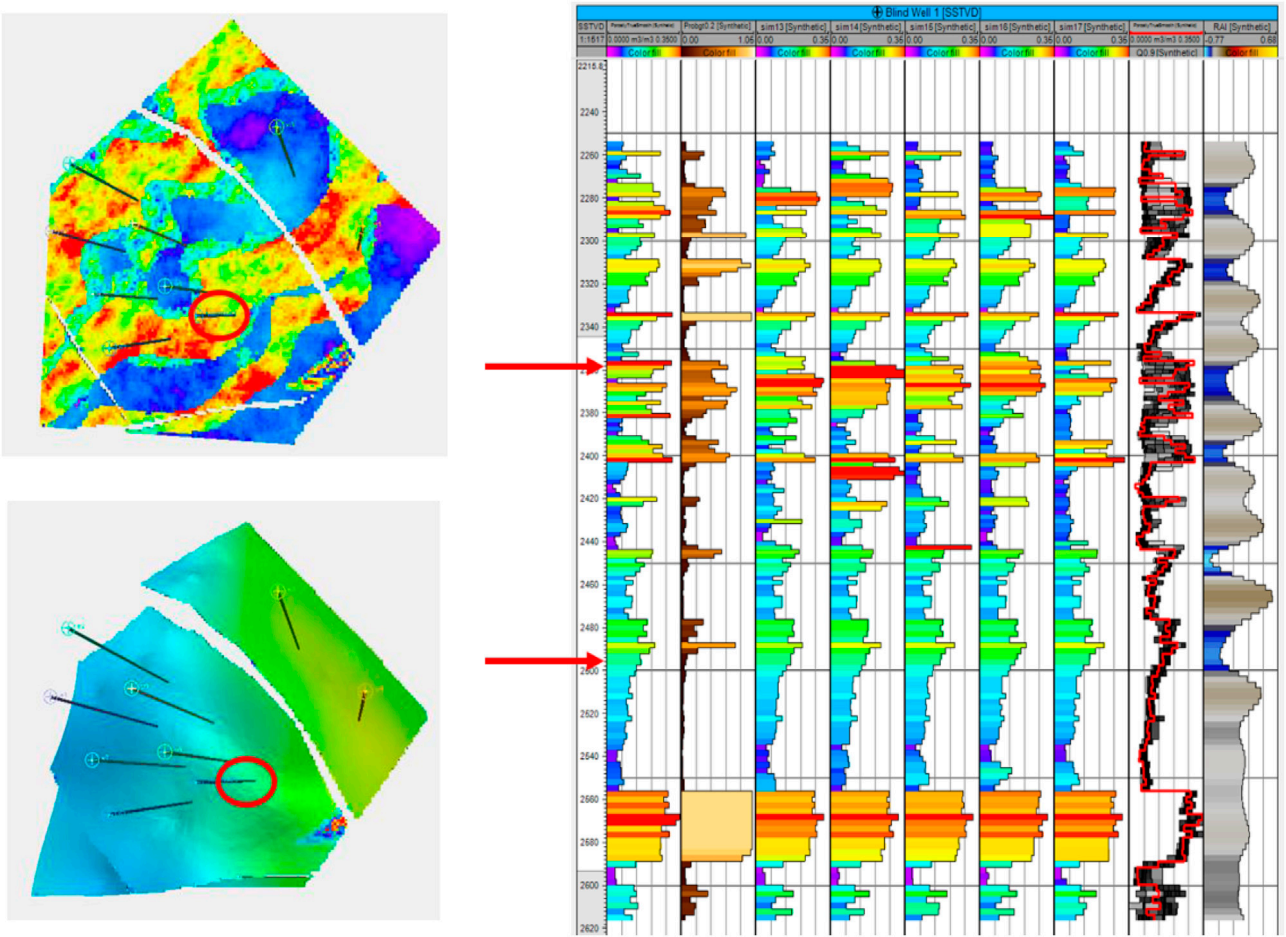
Top left is truth; bottom left is RAI. For the other three columns, the top is eight wells and the bottom 36 wells. Left to right: mean, uncertainty, and simulation from Ember algorithm.

Before showing some numerical results, it is worth noting that the classic Gaussian simulation model was fitted in a few different ways with separate zones, trends, and covariance models. Three different results are shown in Figure 15. It is not claimed that this is the best that can be done with the classic methods, just that considerably more time was spend on these than on the Ember solution and they also used information that would not typically be available in real-world modeling whereas the Ember model did not.

The best Gaussian model was an MM2 model with an unpublished modification developed by the author for the Petrel software suite to account for multiple secondary variables. An MM2 model really amounts to the estimation or simulation of the residuals accounting for the trends (Chiles and Delfiner, 2012). Since the residual variability is quite high and the distribution uses a stationary random function, this accounts for the fact seen earlier and again observed in Tables 1 ,2 that the posterior mean depends strongly on the chosen variogram so that an incorrect choice of variogram can lead to a rapid degradation of estimation and simulation quality. The major advantage of Ember simulation in this case is that it robustly distributes the errors in the right place, assuming that there is enough information to capture heterogeneity in the conditional distributions.

**TABLE 1:** Variance of trror and IQR for three Gaussian models and the Ember model, as well as for the poor choice of “short” variogram for both types. Eight-well case: green is the best case, and red is the worst case.

8 Well model	Zone	Var error	IQR error	Var sim err	IQR sim err
Gaussian 1	Channel	45.20	6.12	71.16	7.28
	Shoreface	15.11	3.80	46.22	7.82
Gaussian 2	Channel	38.85	6.98	81.17	7.67
	Shoreface	10.02	2.63	26.63	5.33
Gaussian 3	Channel	45.03	6.88	80.93	8.11
	Shoreface	25.28	2.52	32.13	3.57
Ember	Channel	39.57	8.07	83.76	7.42
	Shoreface	8.64	2.75	12.52	2.92
Emb short vario	Channel	39.57	8.07	88.61	7.62
	Shoreface	8.64	2.75	21.64	3.92
Gau short vario	Channel	48.51	8.15	95.54	9.39
	Shoreface	20.71	5.66	35.65	7.17

**TABLE 2:** Variance of error and IQR for three Gaussian models and the Ember model, as well as for the poor choice of “short” variogram for both types, 36-well case.

36 Well model	Zone	Var error	IQR error	Var sim err	IQR sim err
Gaussian 1	Channel	32.26	3.38	42.46	5.21
	Shoreface	5.39	1.31	19.13	4.39
Gaussian 2	Channel	28.28	3.98	53.38	6.59
	Shoreface	3.71	0.75	8.93	2.66
Gaussian 3	Channel	31.17	4.11	54.96	5.69
	Shoreface	6.11	1.43	7.15	2.12
Ember	Channel	26.29	4.35	52.67	4.51
	Shoreface	2.56	0.83	3.00	0.96
Emb short vario	Channel	26.29	4.35	52.67	4.45
	Shoreface	2.56	0.83	7.10	1.50
Gau short vario	Channel	43.55	8.21	92.11	8.92
	Shoreface	14.41	4.22	30.83	6.37

**FIGURE 16:**
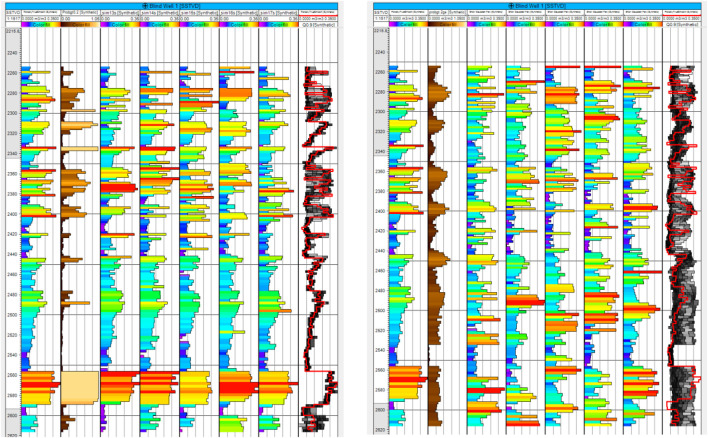
Left is true porosity, while the right-hand side is RAI.

Since the Ember model estimates the conditional distribution at each location, it can be interesting to view what this envelope looks like. To do this, we have selected a location for a blind well and estimated the values there. Results of the Ember model are shown in the eight-well case in Figure 16.

The results show, as expected, that predictions are easier and have lower uncertainty in the shoreface sands. The first track is the true porosity at the blind well location. Each thin black line on the track is a 5% porosity increment. So, porosity values above 15% cross the 3rd black line. The second track shows the estimate of that the location has porosity above 15%. These probabilities are much higher, reflecting greater confidence in the estimate in the shoreface sands. So, for example, there is a thin layer of shoreface sand just above the lower red arrow with porosity just above 15%. It is predicted with high probability (about 0.75) and appears in all five of the simulations. In fact, the simulations vary little within the shoreface and are all very similar to the true porosity log. This is confirmed in view of the envelope itself, which is the second log from the right. It shows the true log in red, overlaying the conditional distribution which is seen as a gray-scale image “spread horizontally” at each depth. The lower bound of the “spread” is the P10, and the upper bound is the P90 of the conditional distribution. The true porosity lies between the P10 and P90 most of the time. The darker the color of the distribution at any point on the track, the higher the probability of having that value (i.e., the color represents probability density). In the shoreface sands, we see that distribution is very concentrated, meaning the conditional distribution has low variability.

In the channel sands, the conditional distribution has got a far larger spread. Interestingly at some depth values, near the top for example (e.g., depth = 2280), the distribution starts very dark on the left, becomes paler in the middle, and returns to dark on the right-hand side. This tells us that the distribution is bimodal at that depth. On reflection, this is not surprising. The prediction knows that it is in a channel interval and within those intervals it must either be in the channel or not. Hence, it has high porosity or low porosity, but only rarely a “middle” porosity. As expected, we see that the true curve switches back and forth between the extremes of the distribution but does not usually fall in the middle. The simulations, since they sample from this conditional distribution, must have the same switching effect; hence, there will be less “shoulder effects” in channel intervals than with a continuous Gaussian model.

Finally in Figure 16, we compare the same well section for the case of the incorrect, short variogram for both the Ember model and the Gaussian case. Since the estimates of probability and the quantiles have not changed, the only difference in the Ember model is the variation in simulations, which is a bit higher. On the contrary, the Gaussian case (calculated a posteriori from hundreds of simulations) shows that the probability estimates are too smooth. The simulations are too random, and the quantiles are far too wide, especially in the shoreface zones.

## Conclusion

There are an increasing number of problems which require estimation of a sparsely observed spatially distributed target variable. In the example given in this paper, we considered estimation of petrophysical variables in a subsurface reservoir. While the target variable is sparse, in most situations, there are many other variables which are covariate with the target. These may be directly observed variables such as those obtained from seismic or electromagnetic measurements, but may also include some variables that can be observed by careful consideration of the environment, including spatial or geological position and proximity to important structural features such as faults. The statistical behavior tends to change locally both per variable and for interactions between variables.

Mathematical models of the spatial phenomena generally need to invoke some notion of stationarity to become tractable. For major studies with significant economic or environmental impact, the user may spend a considerable time on model construction. For three dimensional geological applications, an important part of the art of the practitioner is to segment the model into parts which may be adequately modeled with existing tools. A major objective of the current development is to simplify this workload for the user. The Random Forest algorithm that was used can consume a large number of correlated secondary variables with little overfitting, and it is shown that it can make use of simpler embedded models, provided they have predictive power. Rather than starting with an unrealistic stationary Random Function as prior for the target variable, with the risk that the weakness of the data coupled with the comparative strength of the prior leads to incorrect predictions, this algorithm opts to initially solve a weaker problem. It produces an envelope of distributions at the target locations which depend on the current state of knowledge about the training data and which can subsequently be sampled from to produce realizations of the target variable given the current state of knowledge.

Such a procedure will involve risks. Creating the envelope by resampling means that the model only explores uncertainty within the limits of the observed data. Embedding loses information about the multivariate distribution of the target, so if such information is available, then direct modeling will likely lead to tighter results. Finally, like many spatial modeling algorithms, its performance will degrade when used for extrapolation.

## Data Availability

The project data is available at https://gearth/earthflattener/EmberData.
