# Supplementary material for: An Application of an Embedded Model Estimator to a Synthetic Nonstationary Reservoir Model With Multiple Secondary Variables
Source: Front Artif Intell. 2021 Apr 15;4:624697. doi: 10.3389/frai.2021.624697 (PMC8083000; doi:10.3389/frai.2021.624697)
Supplement: Supplementary file 1 [file presentation1.pdf]

An application of an Embedded Model Estimator to a synthetic non-stationary reservoir model with multiple secondary variables

This is Supplementary Material to the main article which is at https://www.frontiersin.org/articles/10.3389/frai.2021.624697/abstract

Colin Daly

Schlumberger Ltd. 55 Western Avenue, Milton Park, Abingdon, OX14 4RU

Correspondence:
Colin Daly
cdaly@slb.com

Keywords: Geostatistics, Machine Learning, Random Forest, Ember, Spatial Statistics, Reservoir Modelling

A Background

A Decision (or Random) Forest is an ensemble of Decision Trees. Decision Trees have been used for non-parametric regression for many years and their theory is reasonably well understood (Győrfi et al., 2002). They work by inducing a partition of space into hyper-rectangles with one or more data in each hyper-rectangle. To be concrete, suppose we have observations $(Z_i, Y_i), i = 1, \dots, n$ with each Y_i being a vector of predictor variables of dimension p and Z_i the ith training observation of a target variable that we wish to estimate. This paper is concerned with spatial data, so that we are looking for estimation or simulation of $Z(x)$ at a location x. The notation Z_i is thus shorthand for $Z(x_i)$, the value of the target variable at the observed location x_i. The basic idea behind decision trees is to construct the tree using the training data (Z_i, Y_i) and to predict at a new location x by passing the vector $Y(x)$ down the tree reading off the values of Z_i stored in the terminal node hyperrectangle, using them to form the required type of estimator.

A decision tree is grown by starting with all data in the root node. Nodes are split reclusively using rules involving a choice about which of the p coordinates/variables Y^p in Y to use as well as the value of that variable used for the split, s_p, with vectors (Z_i, Y_i) going to the left child if $Y_i^p < s_p$, and to the right child if not. When tree growth terminates (another rule about size of terminal node), the geometry of the terminal node is therefore a hyper-rectangle. The associated set of Z_i are stored in that terminal node. In random forests the choices of variables and splits are randomized in ways explained in the next paragraph, but for now let θ be the set of parameters chosen and call $T(\theta)$ the tree created with this choice of parameters. Denote by R the hyper-rectangles associated with the leaves of the tree and call $R(y, \theta)$ the rectangle which represents the terminal node found by dropping $Y(x) = y$ down the tree $T(\theta)$. As stated before, estimations will be created from these terminal nodes, so for example, the estimate of the mean value of $Z(x)$ given $Y(x) = y$ from the single decision tree $T(\theta)$ is

$$\hat{\mu}(y,\theta) = \sum_{i\epsilon R(y,\theta)} \omega_i(y,\theta) Z_i$$

where the weight is defined by $\omega_i(y,\theta) = \frac{\mathbb{I}_{\{Y_i \in R(y,\theta)\}}}{\sum_j \mathbb{I}_{\{Y_j \in R(y,\theta)\}}}$

i.e., the weight is 1 divided by the number of data in the hyper-rectangle when Y_i is in the terminal node and is 0 when it's not.

Decision Forests are just ensembles of decision trees. The Random Forest is a specific type of decision forest introduced by Leo Breiman (1999, 2004) which have been particularly successful in applications combining the ideas of Bagging (Breiman, 1996) and Random Subspaces (Ho, 1998). In most versions of decision forest, randomization is performed by selecting the data for each tree by subsampling or bootstrapping and by randomizing the splitting. We extend the parameter θ to cover the data selection as well as splitting. An adaptive split makes use of the Z_i values to help optimize the split. A popular choice is the CART criterion (Breiman et al., 1984), whereby a node t is split to $t_l = \{i \in t;\ Y_i^p < s_p,\}$ and it's compliment t_r. The variable p is chosen among a subset of the variables chosen randomly for each split such that the winning split variable and associated split value are the ones that minimize within node variance of the offspring. A slightly more radical split is employed in this paper. Instead of testing all possible split values for each variable, a split value is chosen at random for each variable candidate and then, as before, the within node variances for children calculated and the minimum chosen. This additional randomness seems to help reduce visual artefacts when using a random forest spatially.

The weight assigned to the ith training sample for a forest is $\omega_i(y) = \frac{1}{k}\sum_{i=1}^{k} \omega_i(y,\theta_i)$ where k is the number of trees. The estimator of the conditional distribution, which we need for Ember is then (Meinshausen, 2006)

$$\hat{F}(Z(x) = z|Y = y) = \sum_{i=1}^{n} \omega_i(y)\, \mathbb{I}_{\{Z_i < z\}}$$

And the forest estimate of the conditional mean is $\hat{\mu}(x|y) = \sum_i \omega_i(y) Z_i$.

There is a large literature on Random Forests, so we only mention a few that were helpful to the current work. As well as the foundational papers by Breiman and Meinshausen, Lin and Jeon (2006) describe how they can be interpreted as k-PNN, a generalization of nearest neighbour algorithms and give some good intuitive examples to help understand the role of weak and strong variables, splitting strategies and terminal node sizes. Establishing consistency is relatively easy for Random Forests with some reasonable conditions (we look at an example later). However, there has not of yet been a full explanation of how Breiman's Forest achieves such good results. Nonetheless, some good progress has been made, extending the basic consistency results. The PhD of Scornet (2015) includes a demonstration of the consistency of the Breiman forest in the special case of additive regression models (that chapter was in conjunction with Biau and Vert). Other results concern Central Limit Type results for Random Forests (Athey et al (2019) and references therein). Mentch and Zhou (2019) investigate the role of the regularization implicit in Random Forests to help explain their success.

A.1 Embedding Models in Decision Forests

If we have one or more models, $M_j(x), j = 1, \dots, l,$ which are themselves estimators of $Z(x)$, it is possible to embed these for use within a random forest. As described in the main article, the reason we might want to do this is to make use of information that is not directly available in the form of observed data. In spatial modelling, the variogram or covariance function can give information about the spatial continuity of the variable of interest. A model, such as kriging (Chiles and Delfiner, 2012), which is based on this continuity is well known to be a powerful technique for spatial estimation. In cases where there are a number of other predictor variables (often called secondary variables in the Geostatistics literature), the standard method of combining the information is through linear models of coregionalization (Wackernagel, 2003). The linearity of the relationship between variables can be restrictive and correlations may not be stationary. An alternative is the cloud transform (Kolbjørnsen and Abrahamsen, 2004) which allows a multivariate estimate of a conditional at the target location but does not use any direct interaction with remote target locations until the simulation which uses a P-field method. Probability aggregation (Mariethoz et al., 2009) combines conditional distributions created from the secondary data and from the spatial target data separately. Barnett et al. (2014) use a projection pursuit approach to provide multivariate gaussian transforms for the variables.

It is well known that estimators such as kriging have good properties in their own right (e.g. BLUE) and can be more tolerant of departures from stationarity than associated full multivariate models. These considerations, as well as the possibility of a simplified workflow have prompted the idea of embedding models. The embedded model then acts as an Oracle, which is called to produce estimates at a location, but there is no requirement to concern ourselves with its higher order statistics. So, in this case, rather than try to fit a probability model to the target variable directly, the Ember model captures most of the power of the embedded model while allowing for better modelling of the relationship between variables and of local variability of the target variable. The probability model then emerges as the envelope of distributions $\hat{F}(Z|Y, M\ )$ which doesn't provide an interpretation as a stationary RF for Z as it depends on the current state of knowledge but does allow for exploration of uncertainty through sampling. The embedding is *lazy* both in the sense that rather than looking for potentially very complex interactions between variables, it tries to use the recognized power of Random Forests to deal with this aspect of the modelling and in the sense that no effort is expended on explicitly interpreting Z as some modification of a stationary RF.

In the sort of applications considered here, the Y variable will usually include the spatial location, x, meaning that the forest will explicitly use spatial coordinates as training variables. This means that at each location, we can estimate the conditional distribution $\hat{F}_x \stackrel{\text{def}}{=} \hat{F}(Z(x)|Y(x) = y, M(x) = m\ )$, with Y a vector of size p and M a vector of size l. We call this family of estimates the envelope of Z. Note, this is not the full conditional distribution at x, rather it will be treated as a marginal distribution at x that we will sample from in section 5.3. To emphasize that this is not a model of a spatial random function, we note that the mean of $\hat{F}_x$, $\hat{\mu}_x$, is a trend rather than an exact interpolator, although it generally performs well and will resemble Kriging closely when the latter is a strong variable (Daly 2020) .

Training a forest with embedded models requires an extra step. In many cases the embedded model is an exact interpolator so $M(x_i) = Z_i$. This is the case if the embedded model is kriging. If training were to use this exact value, then the forest would over-train and learn little about how the model behaves away from the well locations, so this is not a useful strategy. A more promising strategy is to use cross validated estimates. Let m_{-i} be the cross validated values found by applying the spatial

model at data location x_i, using all the training data except that observed at x_i. This leads to the training algorithm

1) Choose the iid parameters $\theta_i$for the ith tree.
2) Select a subset of data, the in-bag samples, for construction of the ith tree (either by subsampling or bootstrapping)
3) For each model and each in-bag sample, using all other in-bag samples, calculate the cross validated estimate m_{-i}.
4) Construct the ith tree using (Z_i, Y_i, m_{-i})

Since the model has now been 'converted into' a datum at each location x_i, to keep the notation simple, we will continue to talk about training with (Z_i, Y_i) but need to remember that some of the Y_i are actually embedded models. The effects of this are that

a) each tree is constructed on a different data set (as the embedded models depend on the actual in-bag sample)
b) Since the m_{-i} are not independent, we can no longer make the iid assumption for (Z_i, Y_i) that is usually made for random forests.

A.2 Consistency of the model

Meinshausen (2006) shows that his quantile Random Forest algorithm is consistent in that the estimate of the conditional distribution converges to the true conditional distribution as the number of samples increase.

The assumptions needed for theorem A.1 are

1) Y is uniform on $[0,1]^p$. In fact, it is only required that the density is bounded above and below by positive constants. We discuss what this means for embedded models later.

2) For node sizes,

A) The proportion of observations for each node vanishes for large n (i.e. is o(n)).

B) The minimum number of observations grows for large n (i.e., 1/min_obs is o(1)).

3) When a node is split, each variable is chosen with a probability that is bounded below by a positive constant. The split itself is done to ensure that each sub-node has a proportion of the data of the parent node. This proportion and the minimum probabilities are constant for each node.

4) $F(z|Y = y)$ is Lipschitz continuous.

5) $F(z|Y = y)$ is strictly monotonically increasing for each y.

Theorem 1 (Meinshausen) When the assumptions above are fulfilled, and if, in addition, the observations are independent, the Embedded Forest estimate is consistent pointwise, for each y,

Theorem 1 remains valid when using embedded variables provided the hypothesis hold and the samples can be considered iid. The variables are iid when $U(x) = F(Z(x)|Y(x))$ are iid. While this is not necessarily the case, it is often quite a good approximation and may justify an embedding

approach with general models. The hypothesis is not required when kriging is a strong embedded variable. Theorem 2 (Daly, 2020) is quite simple. It is known that as data becomes dense around a point to be estimated, that kriging converges to the true answer. This just shows that the same is true here when either the embedded variable is kriging and is a strong variable or when the spatial coordinates x,y,z are strong variables. For the demonstration, we need to strengthen assumption 1 and weaken assumption 4. Let $\mathcal{D}$ a closed bounded subset of $\mathbb{R}^n$.

1a) The embedded data variables in Y are uniform on $[0,1]^p$. In fact, it is only required that the density is bounded above and below by positive constants. Moreover the samples occur at spatial locations $S_n = \{x_i\}_{i=1}^n$ such that, for any $\epsilon > 0$, $\exists\, N$ such that $\forall x \in \mathcal{D}, \exists\, x_l \in S_n$ with $d(x, x_l) < \epsilon$ for all $n > N$. In other words, the samples become dense uniformly.

4a) $Z(x), x \in \mathcal{D}$ is a continuous mean squared second order random function with standard deviation $\sigma(x) < \infty$.

Theorem 2 Let $\mathcal{D}$ a closed bounded subset of $\mathbb{R}^n$. Let $Z: \mathcal{D} \to L^2(\Omega, \mathcal{A}, p)$ be a continuous mean squared second order random function with standard deviation $\sigma(x) < \infty$, and if, in addition, assumptions 1a, 2, 3 and 5 hold, then

1) If kriging is an embedded model and it is a strong variable for the Random Forest, then the Ember estimate $\hat{\mu}(x|y) = \sum_i \omega_i(y) Z_i$, converges in L^2 to Z(x).

2) If the random forest also has a mean, $m(x)$, that is continuous in x, is trained on the coordinate vector x, and if each component of x is a strong variable, then the Ember estimate $\hat{\mu}(x|y) = \sum_i \omega_i(y) Z_i$, converges in L^2 to Z(x).

A.3 Simulation

For simulation, we need a full probability model. Defining the sampling variable as $U(x) = F(Z(x)|Y(x) = y)$, the required hypothesis is that the sampling variable is a uniform m.s continuous stationary ergodic random function. This means that the class of random functions that we can model are of type $Z(x) = \psi_x(U(x))$ for some monotonic function ψ_x, which depends on x. To model $Z(x)$ let us further assume that $U(x)$ is generated as the transform of a standard multigaussian random function. So $U(x) = G(X(x))$ with G the cumulative Gaussian distribution function and X(x) is a stationary Multigaussian RF with correlation function $\rho(h)$. This leads to the relation between Z and X,

$$Z(x) = \varphi_x(X(x)) \equiv \psi_x(G(X(x)))$$

This can be expanded in Hermite polynomials (Daly, 2020), which when locations is considered to be a random variable and the expansion is truncated to first order, give equation 3.

To obtain a conditional simulation, it is sufficient to choose a value of $u_i = U(x_i)$ at data location x_i such that the observed target variable data z_i is $z_i = F_{x_i}(u_i)$ where F_{x_i} is the envelope distribution at x_i. Now in general there is more than one such possible u_i, particularly when the envelop distribution is estimated using random decision forests. If fact any $u \in [u_{low}, u_{high}]$will satisfy this condition where u_{low}, u_{high} can be read off the Ember estimate of F_{x_i}. Since in this section, we assume that u is

created as a transform of a Gaussian RF, then a truncated Gaussian distribution can be used for the sampling (e.g. Freulon, 1992). The simulation process becomes,

1) Infer a covariance for the underlying Gaussian X(x) (for example, using equation 3)
2) For each simulation:
    a. Use a truncated gaussian to sample values of u_i satisfying $z_i = F_{x_i}(u_i)$
    b. Construct a conditional Uniform Field U(x) based on X(x) such that U$(x_i) = u_i$
    c. Sample from $F(Z(x)|Y(x) = y)$ using U(x) for each target location x.

Note: The simulation is based on resampling the training data. So, if the sampling is close to uniform (i.e. each sample ends up in a hyperrectangle of approximately the same hypervolume), then the simulation will roughly follow the same distribution as the training data.

References

Athey et al. (2019) Generalized Random Forests, Annals of Statistics Vol 47. No 2. 1148-1178

Barnett, R., Manchuk, J. and Deutsch, C. (2014) Projection Pursuit Multivariate Transform. Math. Geosci. 46:337-359

Breiman L, Friedman J., Stone C., Olshen R.A. (1984) Classification and Regression Trees. Taylor & Francis

Breiman L. (1996) Bagging Predictors. Machine Learning **24,** 123–140 (1996).

Breiman L. (2001) Random Forests. Machine Learning **45,** 5–32 (2001).

Breiman L. (2004) Consistency for a simple model of Random Forests. Technical Report 670. Statistics Dept. Univ. California at Berkeley

Chiles, Jean-Paul and Delfiner, Pierre (2012). Geostatistics. Modelling Spatial Uncertainty. Wiley.

Daly, C (2020) An Embedded Model Estimator for Non-Stationary Random Functions using Multiple Secondary Variables. arXiv:2011.04116 **[stat.ME]**

Daly C., Hardy M., McNamara K. (2020) Leveraging Machine Learning for Enhanced Geostatistical Modelling of Reservoir Properties. EAGE Amsterdam.

Freulon, X. (1992) Conditioning a Gaussian Model with inequalities. Geostatisitcs Troia, Springer, Dordrecht, 1992 pp201-212.

Győrfi, L, Kohler M, Krzyzak A, Walk H (2002) A distribution-free theory of nonparametric regression. Springer-Verlag, New York.

Ho, Tin Kam (1998) The random subspace method for constructing decision forests. IEEE Transactions on Pattern Analysis and Machine Intelligence, vol. 20, no. 8, pp. 832-844, Aug. 1998

Kolbjørnsen, O and Abrahamsen, P (2004) Theory of the Cloud Transform for Applications. Geostatistics Banff, Springer, Dordrecht, 2005, pp45-54

Lin, Yi and Jeon, Yongho (2006) Random Forests and Adaptive Nearest Neighbours. JASA. Vol. 101, No 474. Pp578-590

Meinshausen., N [2006] Quantile Random Forests. J. Machine Learning Research 7, 983-999

Mentch, Lucas and Zhou, Siyu (2019). Randomization as Regularization: A Degrees of Freedom Explanation for Random Forest Success. arXiv:1911.00190v1

Scornet, Erwan, (2015) Learning with Random Forests. PhD . Univ Pierre et Marie Curie.

Wackernagel, Hans (2003) Multivariate Geostatistics. Springer.
